# Cat bite injuries to the hand and forearm: the impact of antibiotic treatment on microbiological findings and clinical outcome

**DOI:** 10.1007/s00402-024-05277-7

**Published:** 2024-03-28

**Authors:** Sebastian Wangler, Miriam Elias, Linus Schoepke, Dominique N. Merky, Rahel Meier, Esther Vögelin

**Affiliations:** 1 Department of Plastic and Hand Surgery, Inselspital, Bern University Hospital, University of Bern, Bern, Switzerland; 2 Department of Orthopaedic Surgery and Traumatology, Inselspital, Bern University Hospital, University of Bern, Bern, Switzerland; 3 Berner Handzentrum, Bern, Switzerland

**Keywords:** Animal bite injuries, Cat bite, Hand, Bite injuries

## Abstract

**Introduction:**

Patients and physicians often underestimate cat bite injuries. The deep and narrow wound seals quickly and provides an environment for the inoculated saliva and bacteria. Interestingly, the literature reports no bacterial growth in the microbiological workup of wound swaps in up to 43%. The time between bite injury and the first clinical presentation, the start of antibiotic treatment and surgical debridement might affect these findings. Therefore, the current project examines if (1) these factors impact the outcome of microbiological results following cat bite injuries and (2) the detection of bacterial growth leads to higher complication rates, longer hospital stays, longer total treatment time, or higher total treatment costs.

**Materials and methods:**

This single-center retrospective study analyzed data from 102 adult patients. All patients received antibiotic and surgical treatment following a cat bite injury. Microbiological samples were collected during surgery in all cases. The time from the bite incident to the first presentation, beginning of antibiotic administration, and surgical debridement was calculated. Demographic data, complication rate, length of hospital stay, total treatment time, and total treatment costs were recorded. (1) A generalized linear model was fitted using the microbiological outcome as the dependent variable. (2) Two groups (negative or positive microbiological results) were formed and statistically compared.

**Results:**

The median age was 50 (SD 16), and 72% were female. (1) The time from the bite incident to the first clinical presentation, antibiotic administration, or surgical treatment was not associated with the outcome of the microbiological result. (2) No significant differences were observed between the two groups.

**Conclusions:**

Our data do not suggest that early antibiotic administration or delayed surgical treatment affects the outcome of the microbiological workup following cat bite injuries to the hand and forearm. The microbiological outcome did not affect the complication rate, treatment time, and total treatment costs.

## Introduction

Animal bite injuries can range from superficial injuries to fatal wounds depending on the animal and affected anatomical location. The lifetime prevalence of animal bites is up to 50%, with dogs and cats causing over 95% of all bite wounds [2, 7, 10]. Animal bites account for about 1% of all visits to emergency departments [8]. Cats have very sharp teeth that penetrate soft tissues and reach deep structures such as tendon sheaths, joints, and bones [3]. The most affected anatomical region is the hand. Cat bite injuries can significantly impact the hand function and ability to work [15, 19, 21]. Patients and physicians underestimate cat bite injuries due to minor cosmetic damage. However, the deep, narrow wound seals relatively quickly and provides an anaerobic environment for the inoculated saliva and bacteria. Significantly delayed treatment can result in severe infections and extended hospital stays [18]. The most common pathogen implicated in animal bites is *Pasteurella multocida,* which can be isolated in up to 90% of healthy cats’ oral cavities [3, 20]. However, in up to 43%, there is no bacterial growth in the microbiological workup of wound swaps following cat bite injuries [11, 15, 17, 19, 21]. Tough, prophylactic antibiotic administration has not been associated with a reduced infection rate after cate bites [14]. The time between bite injury and the first clinical presentation, the start of antibiotic treatment and surgical debridement might affect these findings. Therefore, the current study examines if (1) these factors impact the outcome of microbiological results following cat bite injuries and (2) the detection of bacterial growth leads to higher complication rates, longer hospital stays, longer total treatment time, or higher total treatment costs.

## Methods

The present study represents a single-center retrospective study assessing data of adult patients (> 18 years) with cat bite injuries treated between January 2014 and December 2019. Data represents the same cohort as reported by Schär et al. [17] Inclusion criteria were cat bite injuries to the hand and forearm, primary treatment at our medical center, a microbiological workup, and a complete dataset. Patients with previous surgery in another hospital and cases with incomplete data were excluded. A total of 123 cases were identified. The local ethical committee and the department for research approved the study (KEK-BE: 2018-02076).

The standard treatment pathway for bite injuries includes surgical treatment, antibiotic treatment, and immobilization. The criterion for surgical treatment was the occurrence of one of the following signs or symptoms: severe local pain, erythema extending > 2 cm from the bite, swelling around the bite, secretion of pus, fever, increasing lymphangitis despite conservative treatment (immobilization antibiotic administration, local cooling). All cases were surgically treated, and microbiological samples were cultivated during the surgical debridement.

The following variables were recorded: Demographic data, time from bite incident to first clinical presentation, time to surgery (delay to surgery), time from bite incident to antibiotics administration, time of antibiotic administration before surgery, the total length of antibiotic treatment, complications, duration of hospital stay, incapacity for work, total time of treatment, treatment costs and microbiological outcome (neg/positive), were assessed. Both inpatient care and outpatient service costs were calculated as previously described [17].

Statistical analyses were conducted in R Studio 2022.07.1 + 554 (R v4.0.0), with P 0.05 considered statistically different. Results indicate the mean and the 95% standard deviation (SD). The ‘g*lm’* function was used to fit a linear regression model using the microbiological outcome (neg = 0 and positive = 1 as dependent and the other recorded data points as independent variables.

Patients with positive (proof of bacteria in cultivated surgical samples) and negative microbiological results (no bacterial identification in cultivated surgical samples) were compared. For comparison, an unpaired non-parametric t-test (Wilcoxon matched-pairs signed rank test) was conducted for numeric and a Fisher’s exact test for binary data.

## Results

In the 6-year period, 123 patients with cat bite injuries to the hand and forearm were identified. No microbiological workup was available for twenty patients, and in one patient, the bite location was other than the hand/forearm. This led to the exclusion of 21 cases. In total, 102 cases were identified for further analysis. Two groups were formed. Group I (n = 62) represents all cases with positive bacterial growth in the microbiological workup. Group II (n = 40) represents all cases without bacterial growth in the microbiological workup.

**Fig. 1:**
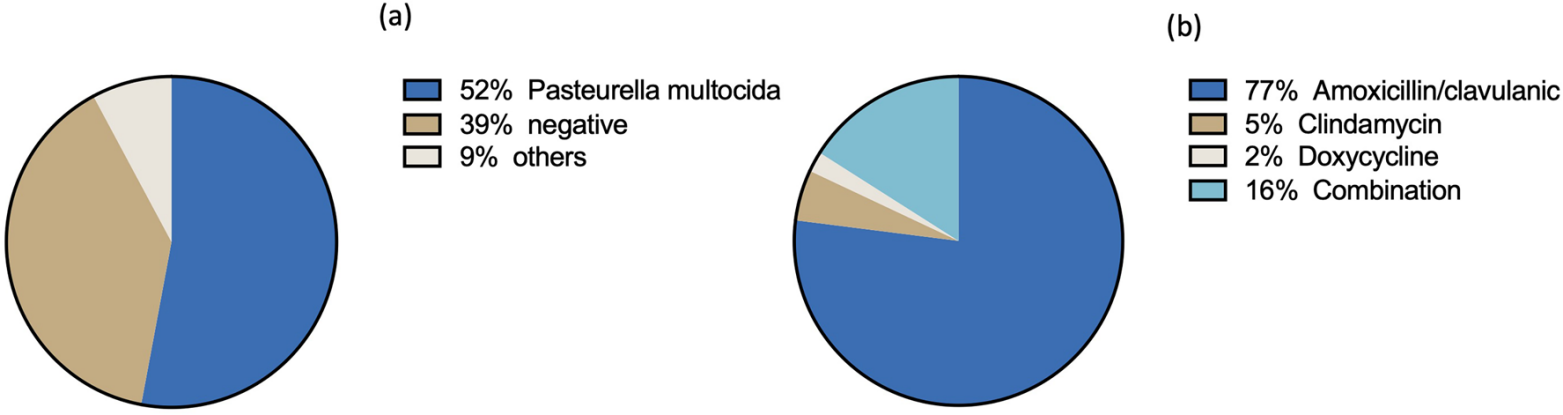
**a** Identified bacterial family and **b** administered antibiotic treatment

The population’s median age was 50 (SD 16), and 73% were female. Of the 102 patients, 90% suffered an isolated injury to the hand, 4% an isolated injury to the forearm, and 6% a combination of hand and forearm. All included patients received antibiotic treatment, 78% amoxicillin/clavulanic, 5% clindamycin, 2% doxycycline, and 16% combined antibiotic therapy (Fig. 1a). Microbiological workup of tissue samples collected during surgery revealed the growth of 53% *Pasteurella multocida*, 2% *Staphylococcus aureus*, 2% *skin flora*, 2% *upper airways flora*, 1% *Neisseria species*, and 1% *Fusobacterium species* together referred as “others” in Fig. 1b. The following data is reported in Table 1: demographic data, time from bite incident to first clinical presentation, time to surgery (delay to surgery), time from bite incident to antibiotics administration, time of antibiotic administration before surgery, the total length of antibiotic treatment, complications, duration of hospital stay, incapacity for work, total time of treatment, treatment costs and microbiological outcome (neg/positive). Nine (9%) patients required a 2nd look revision surgery. Eighty-eight patients suffered from isolated soft tissue infections. Fourteen patients (14%) suffered from at least one of the following complications: Arthritis (n = 7), tendon injury (n = 2), open joint injury (n = 2), phlegmon (n = 1), osteomyelitis (n = 1), complex regional pain syndrome (n = 1), critical tissue loss requiring skin transposition (n = 1), nerve contusion (n = 1).

**Table 1:** Comparison of identified variables among the two groups

	Study population (n = 102)	Groups		
		Group I: positive microbiological workup (n = 62)	Group II: negative microbiological workup (n = 40)	*p *value
Age	50 (SD 16)	50 (SD 17)	49 (SD 15)	0.78
Gender (% female)	73	69	78	0.50
Time from bite incident to the first presentation (days)	1.3 (SD 1.9)	1.3 (SD 1.8)	1.3 (SD 1.9)	0.26
Time from bite incident to antibiotics administration (days)	1.2 (SD 1.9)	1.2 (SD 1.9)	1.3 (SD 1.9)	0.39
Time from bite incident to surgery (days)	3.0 (SD 3.7)	2.7 (SD 3)	3.6 (SD 4.2)	0.25
Begin of antibiotics before to surgery (days)	1.8 (SD 3.2)	1.5 (SD 2.5)	2.3 (SD 4.2)	0.42
Total length of antibiotic treatment	11.7 (SD 8.7)	12.1 (SD 9.9)	11.2 (SD 6.4)	0.85
Complications (%)	*13.7 (n* = *14)*	11.1 *(n* = *7)*	17.5 *(n* = *7)*	0.39
Outpatient treatment (%)	14.3 *(n* = *29)*	27 *(n* = *17)*	30 (*n* = *12*)	0.82
Duration of hospital stay (days)	3.1 (SD 2.2)	3.3 (SD 2.5)	2.8 (SD 1.5)	0.68
Inability to work (days)^a^	36 (SD 76) *(n* = *51)*	41 (SD 96) *(n* = *28)*	30 (SD 41) *(n* = *23)*	0.98
Total time of treatment (days)	43 (SD 68)	47 (SD 80)	37 (SD 43)	0.56
Total treatment costs (CHF)	7232 (SD 8446)	6817 (SD 6293)	7875 (SD 11050)	0.98

^a^ Self-employed and retired patients excluded

A generalized linear model was fitted to test whether one recorded value could predict the microbiological outcome (positive vs. negative). The results are reported in Table 2. None of the evaluated dependent variables significantly predicted the microbiology outcome (neg/positive). The analysis did not reveal significant differences between the two groups (pos. vs. neg. microbiological workup).

**Table 2:** Outcome of the generalized linear model using the microbiological workup (positive vs. negative) as the dependent variable

Independent variable	Odds Ratio	SD error	*p *value	Lower 2.5%	Upper 97.5%
Age	0.99	0.02	0.73	0.96	1.03
Gender	1.99	0.55	0.21	0.68	5.98
Time from bite incident to the first presentation	1.89	0.51	0.21	0.74	5.68
Time from bite incident to antibiotics administration	0.21	1.61	0.34	0.01	7.03
Time from bite incident to surgery	2.00	1.49	0.64	0.07	56.38
Begin of antibiotics before surgery	0.39	1.50	0.53	0.01	10.89
Total length of antibiotic treatment	1.00	0.05	0.97	0.91	1.13
Complications	12.89	1.42	0.07	1.05	301.94
Outpatient treatment	3.06	0.71	0.12	0.77	12.78
Duration of hospital stay	1.09	0.19	0.66	0.75	1.62
Inability to work	0.99	0.01	0.92	0.98	1.02
Total time of treatment	0.99	0.05	0.50	0.99	1.02
Total treatment costs	0.97	0.01	0.90	0.89	1.08

## Discussion

This single-center retrospective study analyzed 102 cases of cat bite injuries to the hand and forearm. All reported cases were treated with antibiotics and surgery, including microbiological workup of collected tissue samples. Bacterial growth was observed in 61% of the analyzed samples. This aligns with the results of other groups identifying bacterial growth in 57–86% of all studied microbiological cultures [4, 11, 15, 19, 21]. With 53% of all positive cases, *Pasteurella multocida* was the predominant identified bacteria in our study cohort. *Pasteurella species* occur in up to 90% of the normal oral flora of cats [11, 15]. The Gram-negative, facultatively anaerobic, and non-spore-forming pleomorphic coccobacillus is known to be one of the major causes of infections following cat bite injuries [1, 16]. Its portion in infected cat bite injuries ranges from 35 to 70% [4, 11, 15, 19, 21]. Primarily the subspecies *Pasteurella multocida* has been associated with a more severe course of infection [6]. An infection with *Pasteurella multocida* usually results in local inflammation and cellulitis within 3–6 h after the bite [13, 20]. The proportion of infections caused by *Pasteurella multocida* (53%) was comparable to other studies with a scale of 35–70% [11, 15, 21].

*Pasteurella multocida* is susceptible to many antibiotics, including amoxicillin-clavulanate, ampicillin, penicillin, tetracycline, azithromycin, chloramphenicol, or doxycycline are a few options available. Importantly, *Pasteurella multocida* is resistant to erythromycin and not sensitive to flucloxacillin and fluoroquinolones [11, 12]. Because of the frequency of mixed infections, including anaerobic bacteria, amoxicillin/clavulanic represents the most recommended first-choice antibiotic treatment for cat-bite injuries [5, 11]. In the reported cohort, 78% of the patients received amoxicillin/clavulanic, followed by clindamycin (5%) and doxycycline (2%). The mean antibiotic treatment time in this cohort was 11.7 days.

In 39% (n = 40) of the reported cases, there was no bacterial growth in the microbiological workup. There are different potential reasons for this finding. The antibiotics or the immune system had already eradicated the bacteria when the sample was taken during surgery. In favor of this explanation, other authors reported that prophylactic antibiotic administration could reduce the infection rate after cat-bite injuries [11]. However, there was no association with antibiotic administration before sample taking and a negative microbiological outcome in our cohort. Secondly, the culture of the *Pasteurella species* is challenging [11]. It is, therefore, possible that the bacterial load on the collected sample was too low to initiate colony formation in tissue culture. Thirdly, sampling error might have occurred when the tissue sample was taken at a location in the wound without bacterial colonization.

Nevertheless, given the local soft tissue inflammation, all patients with a negative microbiological outcome received surgical treatment. The question is whether the enucleated bacteria caused the inflammation of the local soft tissue. As the adaptive immune response takes effect after 4–7 days, the innate immune response is critical in controlling infections during the first period [9]. In the 40 cases with a negative bacterial outcome, the average time from the bite incidence to surgical treatment was 3.6 days (2.7 days in the group with positive bacterial growth). This might indicate that the innate immune response carried out the fight against potentially enucleated bacteria. If this response were adequate, one would expect a positive clinical course without requiring surgical intervention. Surgical treatment in all 40 cases indicates an insufficient defense. However, in this case, one would expect a positive outcome in the microbiological workup. Hence, there might be a different reason for the ongoing local tissue inflammation. It is possible that the bacteria-triggered immune response prolongs following successful eradication or that the bacterial load is too low to be detected but high enough to maintain the inflammatory response. Eventually, agents other than bacteria within the cat’s saliva might also trigger the inflammatory response. Of note, there is no literature supporting this hypothesis.

This study has limitations. We report only cases referred to our hand unit, which does not represent all cat bites in our geographic area during the investigation. Given the inclusion criteria, only cases with available microbiological workup were included. All reported patients received surgical and antibiotic therapy. A group with either isolated surgical or antibiotic treatment would have been ideal as a control to answer the study question. Moreover, we may report severe cases in which a non-surgical treatment was not considered. No data were available to test whether a negative microbiological outcome was associated with less severe clinical cases.

## Conclusions

A positive microbiological outcome was found in the reported cohort in 61%. The most common pathogen was *Pasteurella multocida* (53%), followed by *Staphylococcus aureus* (2%) and *skin flora* (2%). A negative microbiological outcome was not associated with a shorter time of antibiotic administration or a lower frequency of complications. Taken together, our data do not suggest that early antibiotic administration, delayed surgical treatment, or antibiotic administration before microbiological sample collection impacts the outcome of the microbiological workup following cat bite injuries to the hand and forearm. The microbiological outcome did not affect the complication rate, treatment time, and total treatment costs. The local tissue conditions required surgical treatment in all reported cases, including 39% with a negative biological outcome. This might indicate that bacteria are not the only pathogen in cat saliva.

## References

[1] Abrahamian FM, Goldstein EJ (2011). Microbiology of animal bite wound infections. Clin Microbiol Rev.

[2] Amparo ACB, Jayme SI, Roces MCR (2018). The evaluation of animal bite treatment centers in the Philippines from a patient perspective. PLoS One.

[3] Babovic N, Cayci C, Carlsen BT (2014). Cat bite infections of the hand: assessment of morbidity and predictors of severe infection. J Hand Surg Am.

[4] Dire DJ (1991). Cat bite wounds: risk factors for infection. Ann Emerg Med.

[5] Fielding P, Messahel S (2022). Guideline review—human and animal bites: antimicrobial prescribing. Arch Dis Child Educ Pract Ed.

[6] Holst E, Rollof J, Larsson L, Nielsen JP (1992). Characterization and distribution of Pasteurella species recovered from infected humans. J Clin Microbiol.

[7] Holzer KJ, Vaughn MG, Murugan V (2019). Dog bite injuries in the USA: prevalence, correlates and recent trends. Inj Prev.

[8] Jaindl M, Oberleitner G, Endler G, Thallinger C, Kovar FM (2016). Management of bite wounds in children and adults—an analysis of over 5000 cases at a level I trauma centre. Wien Klin Wochenschr.

[9] Janeway CA Jr, TP, Walport M (2001) Immunobiology: the immune system in health and disease, Principles of innate and adaptive immunity, 5th edn. Garland Science, New York. https://www.ncbi.nlm.nih.gov/books/NBK27090/

[10] Khazaei S, Karami M, Veisani Y, Solgi M, Goodarzi S (2018). Epidemiology of animal bites and associated factors with delay in post-exposure prophylaxis; a cross-sectional study. Bull Emerg Trauma.

[11] Kheiran A, Palial V, Rollett R, Wildin CJ, Chatterji U, Singh HP (2019). Cat bite: an injury not to underestimate. J Plast Surg Hand Surg.

[12] Lin H, Liu Z, Zhou Y, Lu W, Xu Q (2022). Characterization of resistance and virulence of *Pasteurella multocida* isolated from Pet Cats in South China. Antibiotics (Basel).

[13] Lloret A, Egberink H, Addie D (2013). *Pasteurella multocida* infection in cats: ABCD guidelines on prevention and management. J Feline Med Surg.

[14] Medeiros I, Saconato H (2001) Antibiotic prophylaxis for mammalian bites. Cochrane Database Syst Rev (2):Cd001738 10.1002/14651858.CD001738 PMC13600173 11406003

[15] Mitnovetski S, Kimble F (2004). Cat bites of the hand. ANZ J Surg.

[16] Oehler RL, Velez AP, Mizrachi M, Lamarche J, Gompf S (2009). Bite-related and septic syndromes caused by cats and dogs. Lancet Infect Dis.

[17] Schär L, Haug L, Vögelin E, Meierb R (2022). Bite injuries to the hand and forearm: analysis of hospital stay, treatment and costs. J Hand Surg Eur Vol.

[18] Seegmueller J, Arsalan-Werner A, Koehler S, Sauerbier M, Mehling I (2020). Cat and dog bite injuries of the hand: early versus late treatment. Arch Orthop Trauma Surg.

[19] Talan DA, Citron DM, Abrahamian FM, Moran GJ, Goldstein EJ (1999). Bacteriologic analysis of infected dog and cat bites. Emergency Medicine Animal Bite Infection Study Group. N Engl J Med.

[20] Westling K, Bygdeman S, Engkvist O, Jorup-Rönström C (2000). *Pasteurella multocida* infection following cat bites in humans. J Infect.

[21] Westling K, Farra A, Cars B (2006). Cat bite wound infections: a prospective clinical and microbiological study at three emergency wards in Stockholm. Sweden J Infect.

